# Retrograde recanalization of radial artery occlusion *via* the distal transradial artery: A single-center experience

**DOI:** 10.3389/fcvm.2022.985092

**Published:** 2022-09-23

**Authors:** Yaowang Lin, Weijie Bei, Huadong Liu, Qiyun Liu, Jie Yuan, Meishan Wu, Xin Sun, Shaohong Dong

**Affiliations:** Department of Cardiology, Shenzhen People's Hospital (The Second Clinical Medical College, Jinan University, The First Affiliated Hospital, Southern University of Science and Technology), Cardiovascular Minimally Invasive Medical Engineering Technology Research and Development Center, Shenzhen Key Medical Discipline, Shenzhen, China

**Keywords:** distal transradial artery, retrograde recanalization, radial artery occlusion, coronary angiography, percutaneous coronary intervention, geriatric disease

## Abstract

**Background:**

Radial artery occlusion (RAO) often occurs after catheterization when using a transradial artery approach.

**Objective:**

This prospective study assessed the success and feasibility of accessing the distal transradial artery (dTRA) for retrograde recanalization of RAO.

**Methods:**

From June 2019 to December 2021, 44 consecutive patients who had undergone cardiac catheterization resulting in RAO were given retrograde recanalization via the dTRA. According to the result of the procedure (primary endpoint), patients' cases were analyzed as successful or failed. Rates of post-operative patency and adverse events were calculated up to 12 months.

**Results:**

The procedural success rate was 88.6%. Compared with the successful group, a significantly higher percentage of patients in the failed group were current smokers and/or suffered from diabetes mellitus (each, 80.0% cf. 33.3%, *P* = 0.046); had undergone at least 3 previous cardiac catheterizations (60.0% cf. 12.8%, *P* = 0.011), lower rate of anticoagulation (30.77% cf. 0%, *P* = 0.048) and exhibited chronic total occlusion (100.0% cf. 51.28%, *P* = 0.041). In each group, one patient each had minor bleeding at the access site and hematoma. The patency rates in the successful group at postoperative 3, 6, and 12 months were 48.7, 43.6, and 35.9%, respectively.

**Conclusion:**

The dTRA approach for retrograde recanalization of RAO showed a high procedural success rate, but with patency rates of <50% at follow-up.

## Introduction

Transradial artery access (TRA) has been used for more than 90% of catheterization procedures with fewer access-related complications, compared with the transfemoral artery approach (1). However, radial artery occlusion (RAO) is a discouraging complication caused by previous cardiac catheterization. The PROPHET study (Prevention of Radial Artery Occlusion—Patent Hemostasis Evaluation Trial) showed that rates of RAO varied from 5 to 12% and 1.8 to 7% at the 24-h and 30-day follow-ups, respectively (2). RAO risks debilitation of the arm, and the TRA cannot be used in further catheterizations.

The distal transradial artery (dTRA) in the anatomical snuffbox is an alternative site for radial artery puncture for cardiac catheterization, including coronary artery angiography (CAG) and percutaneous coronary intervention (PCI) (3). In 2018, Balaban and Elevli (4) showed with 25 patients that it was safe and convenient to perform CAG after retrograde recanalization of RAO via the dTRA. In 2021, Shi et al. (5) also found in a 15-case series that recanalization of RAO through the dTRA was safe and effective. However, the lack of large sample populations and long-term follow-up data have limited these studies.

Accordingly, this prospective clinical study assessed the success rate of retrograde recanalization of RAO via the dTRA approach, investigated possible predictors of failure, and reported long-term results upon follow-up.

## Methods

### Study design and patients

This was a single-center prospective clinical study and was registered in ClinicalTrials.gov (NCT04861389). The institutional review board at Shenzhen People's Hospital approved the study protocol. From June 2019 to December 2021, 44 consecutive patients were included at the Department of Cardiology, Shenzhen People's Hospital, China.

The inclusion criteria were RAO caused by previous cardiac catheterization procedures and accompanied with arm weakness or severe pain. The exclusion criteria were ST-segment elevation myocardial infarction (STEMI) needing primary PCI; without obvious pulsation of the ulnar artery; or cardiogenic shock.

Based on the result of the procedure, for this study analysis the patients were apportioned to a successful group (*n* = 39) or failed group (*n* = 5).

### Radial artery recanalization procedure

**Figure 1:**
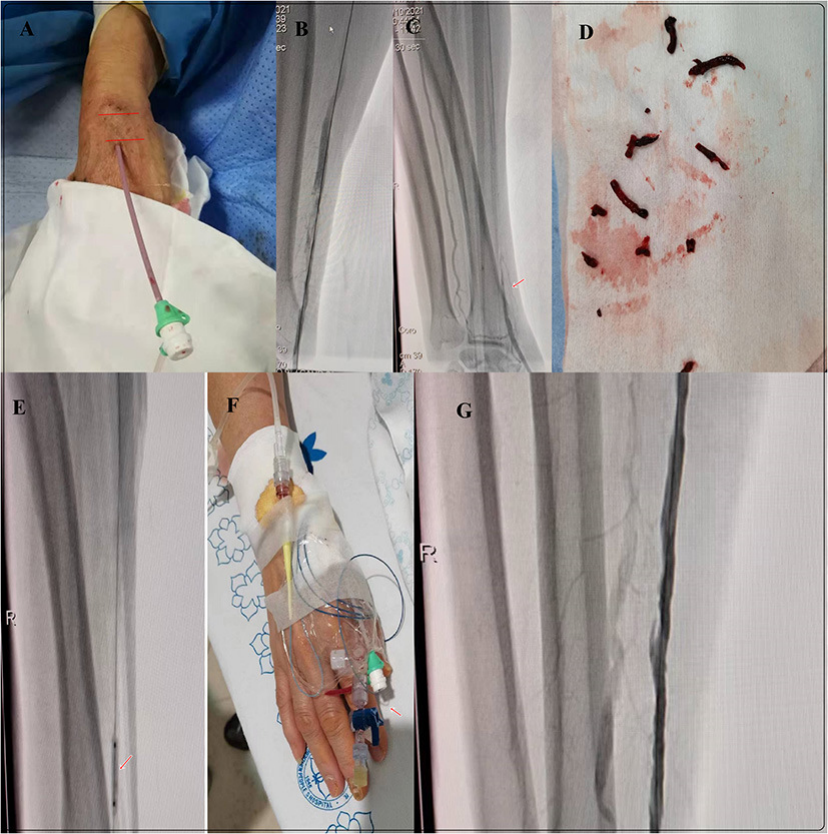
Radial artery recannulation of RAO. **(A)** Insertion of the 6 French introducer sheath via snuffbox with ~1–2 cm depth. **(B,C)** Radial angiography to determine the anatomy of the radial artery, the occluded end, thrombotic load, and the collaterals. **(B)** Acute occlusion. **(C)** Chronic occlusion. **(D)** Aspiration of the thrombus directly through the introducer sheath or aspiration catheter for acute occlusion or chronic occlusion with high thrombotic load. **(E)** Balloon angioplasty of the RAO. **(F)** Thrombolytic therapy with urokinase via finecross microcatheter (Terumo, Japan). **(G)** Final result of the radial angiography.

All the catheterization procedures were performed by a doctor who was experienced in the dTRA approach. The snuffbox access site was punctured by an intravenous catheter needle (1.02 mm). After successful puncture, an introducer sheath (Terumo Medical; Figure 1A) was inserted to a depth of ~1- to 2-cm. Radial angiography was performed to characterize the anatomy of the radial artery, the occluded end, thrombotic load, and the collaterals (Figures 1B,C). For acute occlusion or chronic occlusion with high thrombotic load, aspiration of the thrombus directly through the introducer sheath (7 French sheath is more suitable) or assisted by aspiration catheter was attempted (Figure 1D). If aspiration was unsuccessful or the radial artery angiography showed insufficient restoration after aspiration, balloon angioplasty was performed repeatedly with a 0.014 guidewire or a 0.025 guidewire assist if necessary (Figure 1E). Continuous thrombolytic therapy with urokinase via finecross microcatheter (Terumo, Japan) was given for 6–12 h after intervention surgery in patients with acute occlusion or chronic occlusion with high thrombotic load (Figure 1F). Finally, a satisfactory result on the radial angiography was achieved (Figure 1G).

Before patient discharge and at 3, 6, and 12 months, the patency of the radial artery was evaluated using Doppler ultrasound (Handydop Pro, Medisound Medical Apparatus).

### Study endpoints

The primary endpoint was the success rate of the retrograde recanalization of the RAO, defined as blood flow restoration, monitored by Doppler ultrasound. Secondary endpoints included possible predictors of failure, complications, and patency at follow-up.

### Statistical analysis

The data were analyzed using IBM SPSS 22.0 software. Continuous data are shown as mean ± standard deviation or counting data and percentage. The differences between groups of continuous variables subject to normal distribution were tested by *t*-test. Continuous variables without normal distribution were analyzed by Kruskal Wallis test or Mann-Whitney *U* test. The chi-squared test or Fisher's exact test was used for categorical variables. A difference of *P* < 0.05 was considered statistically significant.

## Results

### Procedural success rate and baseline characteristics

The rate of procedural success was 88.6%. Thus, the successful and failed groups consisted of 39 and 5 patients, respectively (Table 1). Compared with the successful group, a significantly higher percentage of patients in the failed group were current smokers and/or suffered from diabetes mellitus (each, 80.0% cf. 33.3%, *P* = 0.046); had undergone at least 3 previous cardiac catheterizations (60.0% cf. 12.8%, *P* = 0.011); lower rate of anticoagulation (30.77% cf. 0%, *P* = 0.048) and experienced chronic total occlusion (100.0% cf. 51.28%, *P* = 0.041). No other significant differences were found between the 2 groups.

**Table 1:** Baseline characteristics of the dTRA and TRA groups^*^.

	**Successful**	**Failure**	***P***
Subjects*, n*	*N* = 39	*N* = 5	
Age, y	58.26 ± 12.70	66.00 ± 11.77	0.228
Males*, n*	25 (64.19)	3 (60.00)	0.986
Body mass index, kg/m^2^	24.73 ± 3.35	25.51 ± 3.26	0.635
SBP at the cath lab, mmHg	132.20 ± 22.26	127.23 ± 23.29	0.660
Heart rate at the cath lab, bpm	67.20 ± 15.09	81.05 ± 17.97	0.108
Hypertension*, n*	24 (61.54)	3 (60.00)	0.948
Diabetes mellitus*, n*	13 (33.33)	4 (80.00)	*0.046*
Current smoking*, n*	13 (33.33)	4 (80.00)	*0.046*
Previous cardiac catheterization	32 (7.11)	33 (7.33)	0.980
No.1*, n*	30 (76.92)	1 (20.00)	*0.009*
No.2*, n*	4 (10.26)	1 (20.00)	0.523
No≥3*, n*	5 (12.82)	3 (60.00)	0.011
Status			
ATO*, n*	19 (48.72)	0 (0)	*0.041*
CTO*, n*	20 (51.28)	5 (100.00)	
Time from RAO to	37.64 (2.00, 30.00)	206.4	*0.0188*
recanalization, *d*		(60.50, 329.00)	
eGFR, ml/(min.1.73 m^2^)	76.67 ± 18.32	75.28 ± 31.60	0.890
LVEF, %	52.80 ± 3.56	53.16± 13.18	0.893
Diagnosis			
STEMI*, n*	8 (20.51)	3 (12.44)	0.137
NSTEMI-ACS*, n*	6 (15.38)	0 (35.56)	
SCAD*, n*	25 (64.10)	5 (40.00)	
Medical treatment			
Aspirin*, n*	39 (100.00)	5 (100.00)	0.601
Clopidogrel*, n*	9 (23.08)	1 (20.00)	0.922
Ticagrelor*, n*	30 (76.92)	4 (80.00)	
Anticoagulation*, n*	12 (30.77)	0 (0)	*0.048*

* Reported as n (%), unless indicated otherwise.

SBP, systolic blood pressure; ATO, acute total obstruction; CTO, chronic total obstruction; RAO, radial artery obstruction; ACS, acute coronary syndrome; APT, antiplatelet therapy; eGFR, estimated glomerular filtration rate; STEMI, ST-segment elevation myocardial infarction; NSTEMI, non-ST-elevation myocardial infarction; SCAD, stable coronary artery disease; LVEF, left ventricular ejection fraction.

### Procedure and post-procedure characteristics in the successful and failed groups

There were no significant differences between the group regarding procedural or post-procedural characteristics, except for angioplasty (Table 2). The procedure time varied from 15 to 66 min in the successful group. Thirty-seven (84.09%) patients in the successful group underwent CAG or PCI immediately after successful retrograde recanalization of the radial artery. One patient in each group had minor bleeding at the access site and in each group one patient experienced hematoma.

**Table 2:** Procedural and post-procedural characteristics of the successful and failure groups^*^.

	**Successful**	**Failure**	***P***
	***N*** **= 39**	***N*** **= 5**	
Procedural characteristics			
Procedure time, min^**^	43.79 ± 9.40	50.40 ± 5.81	0.064
Guide wire			
0.014	22 (56.41)	5 (100)	0.052
0.025	20 (51.28)	4 (80.0)	0.230
Assist with balloon	35 (89.74)	5 (100)	0.458
Intervention treatment			
Aspiration	17 (43.59)	0 (0)	0.067
Angioplasty	37 (94.87)	0 (0)	*0.000*
Post-procedural characteristics			
Total cost of procedure, USD	336.12 ± 12.45	331 ± 16.44	0.768
Access site minor bleeding	1 (2.56)	1 (20.0)	0.082
Hematoma	1 (2.56)	1 (20.0)	0.082

* Data reported as n (%) unless indicated otherwise.

** Access time was the time from the subcutaneous local anesthetic to the administration of heparin.

### Patency rate at follow-up

**Figure 2:**
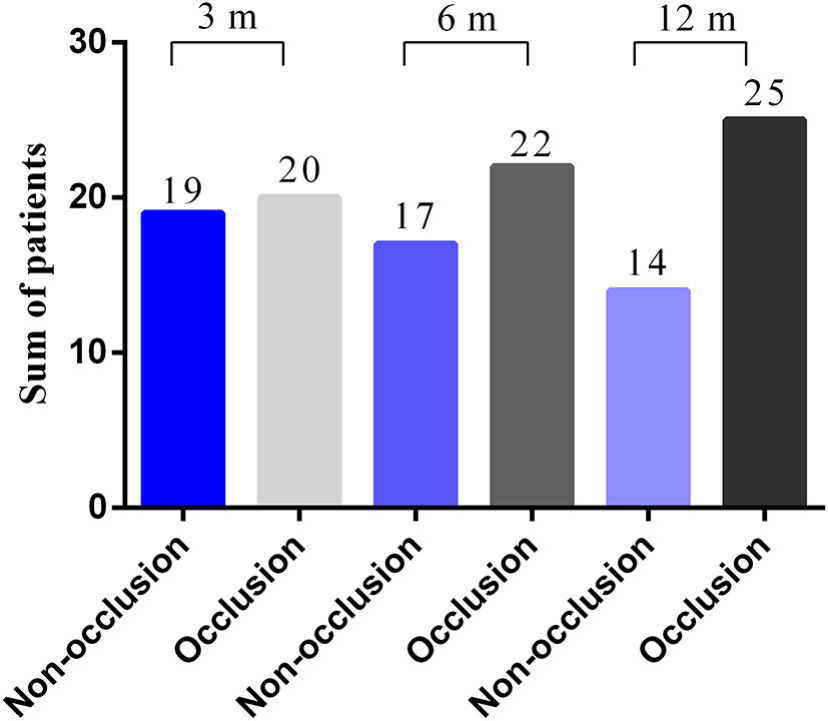
Patency rates at follow-up. The patency rates at 3, 6, and 12 months were 48.72, 43.59, and 35.90%, respectively.

Restoration of the blood flow of the radial artery was monitored by Doppler ultrasound once a month after discharge from the hospital. In the successful group, at the 3-, 6-, and 12-month follow-ups, the patency rates were 48.7, 43.6, and 35.9%, respectively (Figure 2).

## Discussion

The main finding of this prospective clinical study was the high acceptable success rate of the dTRA approach for retrograde recanalization of RAO caused by previous cardiac catheterization. Thus, the dTRA approach is considered feasible for this purpose. However, at the 12-month follow-up, the patency rate was low, <50%.

RAO is a common complication of the TRA approach, with an incidence that varies from 0.8 to 38% (6, 7). Factors related to RAO include body mass index, diabetes, sheath size, anticoagulant usage, and hemostasis (6, 8). Although severe hand ischemia is rarely caused due to the dual vascular supply of the palm arch, RAO still affects the physical activity of the arm or causes severe pain from acute occlusion. RAO also precludes the radial approach from PCI or CAG, although the femoral artery can be used as an alternative, increasing access complications and with less surgical comfort. Finally, RAO in patients under hemodialysis precludes the chance for a spare artery for the formation of fistula.

The dTRA has served as an alternative access for cardiac catheterization since 2017, and its potential advantages have attracted the attention of cardiologic interventionalists (3, 9, 10). Some studies have focused on the dTRA to recanalize RAO in cardiac catheterization. In 2018, Sheikh et al. (11) reported a successful angioplasty via dTRA in recanalizing the left RAO of a 75-year-old man with a medical history of coronary artery bypass, previous multiple PCIs, and severe peripheral vascular disease. Li et al. (12) presented a case of RAO after emergency CAG and PCI in which recanalization was successfully performed through dTRA access. Additionally, case series have suggested that dTRA was safe and convenient for retrograde recanalization of RAO, with success rates of 88 to 93% (4, 5). In the present study, the success rate was similar at 88.6%. The high success rate is important to maintain of radial access for future procedures. Keeping the radial artery patency not only improves the physical activity of the arm, but also reduces procedure complications such as severe haematoma caused by puncturing the femoral artery. Besides, successful RAO recanalisation by dRA is a life-saving option for patients with current or future renal failure who are either on or may be on dialysis. Retaining radial patency may spare one access more for a patient who might be a future candidate for a dialysis fistula, probably in the contralateral arm.

In this study, compared with the successful group, the failed group had a significantly higher percentage with diabetes mellitus, current smokers, chronic total occlusion, and 3 or more previous cardiac catheterizations. These factors have been associated with radial vasospasm, calcification, and poor collateral circulation.

The most common complication of the procedure has been hematoma during balloon angioplasty (4, 13). The following points should be noted carefully. First, the operator should be experienced in dTRA, and especially with a high rate of successful access. Doppler ultrasound can accurately guide the dTRA puncture and improve the success rate of puncture (14, 15). Secondly, the sheath should not be inserted too deeply, but advanced to a depth of 1–2 cm only. If there is no pulsatile blood flow gushing out of the cannula, we should realize that the sheath may be inserted too deep with the occluded segment or thrombus, instead of vascular dissection. Finally, balloon-assisted tracking technology can be applied to navigate the guidewire through the occlusion (5).

The long-term result of retrograde recanalization of RAO remains unclear. No stent was used to achieve patency of the radial artery when undergone dTRA recanalization in this study. First, most radial arteries are around 2.5 mm in diameter and are not suitable for stent treatment. Second, stent for the radial artery interferes with re-puncture. Balaban and Elevli (4) showed that for 14 patients given drug-coated balloon (DCB) treatment after angioplasty, the patency rate was only 33.4% at the 1-month follow-up. In our study, the patency rates at 3, 6, and 12 months were 48.72, 43.59, and 35.90%, respectively. These rates were higher than that of the previous study, and no DCB was used. It may be that DCB treatment is not suitable for RAO. This interesting result may be due to the different mechanisms underlying artery stenosis. DCB was proved effective and durable for preventing restenosis in atherosclerotic disease, but not in a dysfunctional dialysis circuit or RAO caused by repeated punctures (16, 17). Based on the low patency at follow-up, retrograde recanalization of RAO is best for patients who consequently require CAG or PCI immediately. Additionally, the cost of the procedure is much less, and the surgical materials are often needed for subsequent cardiac catheterization. However, for those patients who do not require cardiac catheterization immediately and within 1 year, retrograde recanalization of RAO may not be beneficial for preserving the patency of the radial artery ascribed to the low patency rate with follow-up.

The main limitation of the present study is the small number of patients in the unsuccessful group. No multivariate logistic analysis was conducted to determine possible predictors of failure. In the future, DCB should be compared with conventional balloon angioplasty for RAO. In addition, parameters derived from the Doppler ultrasound were not considered, including diameters of the radial artery before and post-procedure. Finally, this study was performed in a single geographic region. The results warrant larger randomized controlled trials for confirmation.

## Conclusion

This study highlighted the feasibility of the dTRA approach for retrograde recanalization of RAO, with a high procedural success rate but low patency rate at follow-up.

## Data availability statement

The raw data supporting the conclusions of this article will be made available by the authors, without undue reservation.

## Ethics statement

The studies involving human participants were reviewed and approved by Shenzhen People's Hospital. The patients/participants provided their written informed consent to participate in this study.

## Author contributions

YL and WB designed, collected, analyzed and wrote this manuscript, and performed the research. YL, XS, and SD was the principal investigator. All authors contributed to the article and approved the submitted version.

## Funding

This study was supported by Shenzhen Key Medical Discipline Construction Fund (No. SZXK003), Sanming Project of Medicine in Shenzhen (No. SZSM201412012), Shenzhen Foundation (JCYJ20210324113614038), and Guangdong Medical Research Foundation Project (B2020069).

## Conflict of interest

The authors declare that the research was conducted in the absence of any commercial or financial relationships that could be construed as a potential conflict of interest.

## Publisher's note

All claims expressed in this article are solely those of the authors and do not necessarily represent those of their affiliated organizations, or those of the publisher, the editors and the reviewers. Any product that may be evaluated in this article, or claim that may be made by its manufacturer, is not guaranteed or endorsed by the publisher.
